# Effect of Humic Acid on the Growth and Metabolism of *Candida albicans* Isolated from Surface Waters in North-Eastern Poland

**DOI:** 10.3390/ijerph19159408

**Published:** 2022-07-31

**Authors:** Adam Cudowski, Anna Pietryczuk, Andrzej Górniak

**Affiliations:** Department of Water Ecology, Faculty of Biology, University of Białystok, Ciołkowskiego 1J, 15-245 Bialystok, Poland; annapiet@uwb.edu.pl (A.P.); hydra@uwb.edu.pl (A.G.)

**Keywords:** humic acid, *C. albicans*, proteins, sugars, antioxidant enzymes

## Abstract

The aim of this study was to determine the effect of humic acid on the growth and metabolism of *Candida albicans*, a common waterborne pathogenic yeast. At 10–20 mg/L, humic acid caused the greatest increase in biomass and compactness of proteins and monosaccharides, both in cells and in extracellular secretion of the yeast. At higher humic acid concentrations (40–80 mg/L), *C. albicans* cells still had higher protein levels compared to control, but showed reduced levels of metabolites and inhibited growth, and a significant increase in the activity of antioxidant enzymes, indicating a toxic effect of the humic acid. The increase in protein content in the cells of C. albicans combined with an increase in the activity of antioxidant enzymes may indicate that the studied yeast excels in conditions of high water enrichment with low availability of organic matter. This indicates that *Candida albicans* is capable of breaking down organic matter that other microorganisms cannot cope with, and for this reason, this yeast uses carbon sources that are not available to other microorganisms. This indicates that this fungus plays an important role in the organic carbon sphere to higher trophic levels, and is common in water polluted with organic matter.

## 1. Introduction

Humic substances enter surface waters with catchment runoff and can also form autochthonously in waters as a result of physicochemical (pH, oxygen, temperature) and biological factors (bacteria and fungi) acting on dead plant remains [1]. There are three classes of humic compounds: water-soluble fulvic acids and humic acids, and insoluble humins. The chemical structures of humic substances contain functional groups, such as carboxyl, hydroxyl or ketone groups, which can react with living organisms [2]; the effect depends on the concentration and structure of the humic substance [1,3,4]. Humic substances have been shown to primarily interact with cell membranes, causing changes in their permeability, and to modify the activity of enzymes associated with biotransformation. Moreover, these substances have a defensive function against environmental stress factors by inducing the synthesis of stress proteins, such as chaperones and heat shock protein Hsp70, and by increasing the activity of antioxidant enzymes. At high concentrations, humic substances can act as stress factors, causing lipid peroxidation and even triggering carcinogenesis [1,3,4]. The main consumers of dissolved organic matter are bacteria, whose role and place in the microbial loop is already fairly well understood [5,6,7,8]. However, studies conducted in recent years indicate that fungi may be an important link in the biotransformation processes of organic matter. First of all, they play an active part in the microbial decomposition of a variety of organic matter [9,10,11], especially those that are difficult to access for other microorganisms, i.e., lignin, cellulose, hemicellulose, humic substances and various xenobiotics [12,13,14,15,16]. In addition, fungi have been shown to be actively involved in the biotransformation of xenobiotics such as pharmaceuticals, pesticides [17], and heavy metals [18] in aquatic environments, and thus may contribute to the mitigation of anthropogenic stresses and improve the water quality. The prevalence of *Candida albicans* in waters, and its virulence factors and pathogenicity, have led to recent proposals that this yeast should be regarded as a biological indicator of water quality [19,20,21,22]. However, it is necessary to determine the influence of organic matter on the metabolism of this yeast.

The aim of this study was to determine the effect of different concentrations of humic acid on the growth of the potentially pathogenic yeast *Candida albicans*, the content of basic metabolites (proteins, monosaccharides) both in its cells and in its extracellular secretions, and whether humic acid induces oxidative stress in yeast cells, as shown by the activity of antioxidant enzymes in fungal cells. The experiment used a number of humic acid concentrations within a range of 5–40 mg/L (55–60% C), corresponding to the range that occurs naturally in surface waters, as well as one much higher concentration (80 mg/L) [23].

## 2. Material and Methods

### 2.1. Material and Growth Conditions

The experiment was conducted using the yeast *C. albicans* (EF192231.1), which was most commonly isolated from limestone and lotic waters of NE Poland. The isolated fungi species were ranked using the Sanger sequencing method with two primers, ITS1 (5′-CTTGGTCATTTAGAGGAAGTAA-3′) and ITS4 (5′TCCTCCGCTTATTGATATGC-3′). The sequence products were analyzed with BLAST through the National Center for Biotechnology Information (NCBI) website by aligning input sequences against published nucleotide sequences, with a coverage rate of 99.9%.

Pure colonies of the fungi identified to the species level were recultured in malt extract agar medium (1% malt extract, 1.5% agar) (Biocorp, Issoire, France). For the purpose of establishing experimental cultures, the fungi were transferred to sterile disposable culture bottles containing 200 mL of liquid medium previously sterilized by autoclaving (1% malt extract, pH 5.0) and enriched with humic acid or without the addition of humic acid (control culture). The control culture was to eliminate the influence of the medium on the yeast metabolism. 

The determination of fresh fungal mass involved weighing the nitrocellulose filters 0.22 µm (Millipore, Burlington, MA, USA) on an analytical balance and then filtering the suspension through the filters. After the procedure, the filters were dried at room temperature and weighed again.

Prior to establishment of the experimental culture, *Candida albicans* (EF192231.1) was cultured on a sterile substrate of malt extract agar at 37 °C and then transferred to liquid medium and incubated at 25 °C for 2 weeks. From this, 6 sterile plastic bottles were filled with liquid medium. Humic acid (Sigma Aldrich, St. Louis, MO, USA) at various concentrations was added to 5 of the bottles: 5 mg/L, 10 mg/L, 20 mg/L, 40 mg/L, 80 mg/L. The control sample was the one bottle without humic acid. All samples were then incubated at 25 °C for 5 days, and the cultures filtered through 0.20 µm nitrocellulose filters (Millipore) and centrifuged at 2500 rpm for 10 min [24]. All parameters were measured using a Shimadzu UV-Vis 1201 spectrophotometer (Kyoto, Japan). The experiment was repeated four times in three repetitions. All statistical analyses were made in SPSS19 using Kruskal–Wallis tests. The standard deviations of the average of all parameters were below 5%. The results are presented as average values ± SD.

### 2.2. Determination of Proteins in Biomass and Secretion of Candida albicans

Filtered fresh biomass of *Candida albicans* was homogenized in liquid nitrogen with 1 mol/L NaOH solution to isolate proteins from the fungal cells. The supernatant was used for protein determination in the culture medium. Filtered fresh mass of fungi was transferred to a boiling water bath for 10 min. Next, the samples were centrifuged at 2500 rpm for 10 min [24]. The supernatant was transferred to tubes and suitable reagents were added, such as distilled water, Folin reagent, five-water copper(II) sulfate solution, NaOH solution, and sodium carbonate solution. Then, the blank trial (1 mol/L NaOH solution) and benchmark (0.03% albumin solution) were conducted. Prepared samples were used for the determination of protein in the biomass and secretions by measuring spectrophotometric absorbance at λ = 750 nm. The quantities of protein biomass and secretions of *Candida albicans* were presented as milligrams per gram fresh weight following Lowry’s method [25]. 

### 2.3. Determination of Monosaccharides in the Biomass and Secretions of Candida albicans

Monosaccharides in the *C. Albicans* biomass and secretions were determined according to Samogyi–Nelson methods. Filtered samples were homogenized in liquid nitrogen with 96% ethanol to isolate monosaccharides from the fungal cells. The supernatant was used for monosaccharide determinations in the culture medium. Filtered fresh mass of fungi was transferred to a water bath at 70 °C. After 20 min the samples were centrifuged at 2500 rpm for 10 min. Blank (ethanol) and benchmark (ethanol glucose) solutions were then prepared. A mixture was prepared with sodium sulfate, potassium sodium tartrate, sodium bicarbonate, sodium carbonate, and five-water copper (II) sulfate and sodium sulfate were added. All samples were transferred to the water bath for 20 min, then centrifuged at 2500 rpm for 10 min. The reaction mixture consisted of seven-water sodium metaarsenate, seven-water ammonium molybdate, distilled water and sulfuric acid (VI). Samples were incubated for 5 min at room temperature, and absorbance measured spectrophotometrically at λ = 540 nm. The quantity of monosaccharides in the biomass and secretions of *Candida albicans* were presented as milligrams per gram fresh weight [26,27]. 

### 2.4. Determination of Catalase (CAT) Activity in Candida albicans Biomass

Catalase activity was determined after biomass samples were homogenized in liquid nitrogen with phosphate buffer (pH 6.0) and centrifuged at 2500 rpm for 10 min. The benchmark consisted of phosphate buffer, distilled water and hydrogen peroxide [28]. Catalase activity was determined by means of mixtures with phosphate buffer, yeast extract and hydrogen peroxide. Reduction of H_2_O_2_ was monitored by a decrease absorbance at λ = 240 nm after 1 and 5 min. The resulting absorbances were calculated and set out as units of enzyme activity per milligram of protein. An enzyme activity unit was defined as the quantity of enzyme needed to catalyze a reduction of 1 mmol/L H_2_O_2_ in 1 min at 30 °C.

### 2.5. Determination of Superoxide Dismutase (SOD) Activity in Candida albicans Biomass

Superoxide dismutase activity was determined after biomass samples were homogenized in liquid nitrogen with anhydrous magnesium sulfate dissolved in phosphate buffer with dithiothreitol (DTT) and two-water disodium versenate. Next, the samples were centrifuged at 2500 rpm for 10 min. Supernatant was transferred to 3 empty tubes and to another 3 tubes filled with distilled water. To all tubes phosphate buffer, riboflavin solution, methionine solution, and nitro blue tetrazolium chloride (NBT) were added. The benchmark was a single sample with yeast extract mixed with distilled water, incubated in the dark for 20 min at room temperature. Another two samples were filled with distilled water and two others with yeast, and incubated in the light for 20 min at room temperature. After this time, all samples were measured for spectrophotometric absorbance at λ = 560 nm. These methods [29] allow the determination of the quantity of superoxide dismutase by determining the reduction of the nitro blue tetrazolium chloride (NBT). The results were set out in units of enzyme activity per milligrams of protein. A unit of enzyme activity was defined as the quantity of enzyme needed to catalyze a 50% reduction of NBT in relation to the control reaction.

The quantities of protein and monosaccharide biomass and secretions of *Candida albicans* are presented as milligrams per gram fresh weight. The catalase and SOD activity was set out in units of enzyme activity per milligram of protein.

## 3. Results

**Figure 1:**
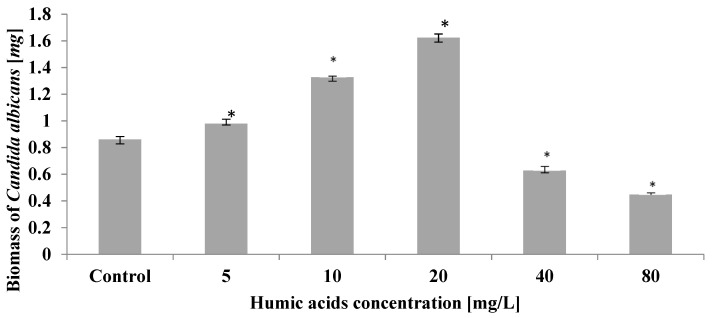
The effect of humic acids on biomass of *Candida albicans* on the 5th day of culture (*n* = 8, average values ± SD, (*p* ≤ 0.01), (*) shows statistically significantly different values in comparison with control sample.

The cultures in which exogenous humic acid was applied at 10 mg/L and 20 mg/L showed the highest statistically significant (*p* ≤ 0.01) increase in *Candida albicans* biomass, by 55% and 88%, respectively, compared to control. At humic acid concentrations of 40 mg/L and 80 mg/L, the yeast biomass decreased by 27% and 48%, respectively, compared to control (Figure 1).

**Figure 2:**
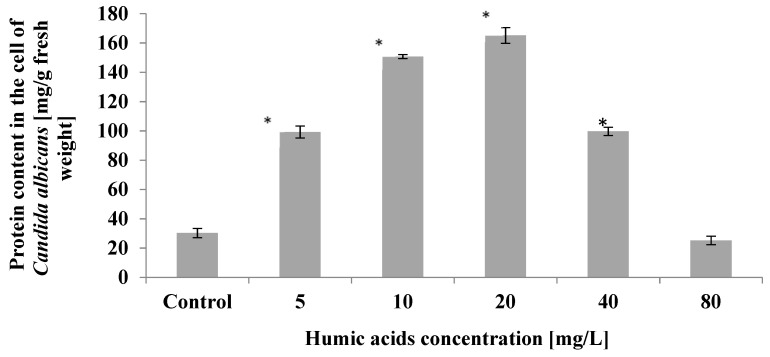
The effect of humic acids on protein content in cell of *Candida albicans* on the 5th day of culture (*n* = 8, average values ± SD, *p* ≤ 0.01), (*) shows statistically significantly different values in comparison with control sample.

The analyses showed a statistically significant (*p* ≤ 0.01) increase in protein content in *Candida albicans* cells growing in the presence of humic acid at concentrations ranging from 5 mg/L to 40 mg/L. The highest protein content in yeast cells was observed at humic acid concentrations of 20 mg/L. The content of these metabolites then increased by 5 times compared to control (Figure 2).

**Figure 3:**
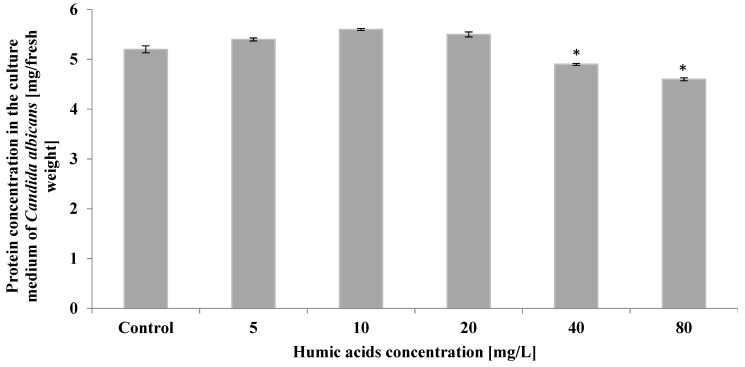
The effect of humic acids on the concentration of protein in the culture medium of *Candida albicans* on the 5th day of culture (*n* = 8, average values ± SD, *p* ≤ 0.01), (*) shows statistically significantly different values in comparison with control sample.

Humic acid had only a small effect on the protein content of *Candida albicans* secretions. At 5 mg/L, 10 mg/L, and 20 mg/L, the protein concentration in the culture medium remained at the control level (a statistically insignificant increase), while at 40–80 mg/L, a 6–12% decrease in secretion protein concentration was evident compared to control (Figure 3). 

**Figure 4:**
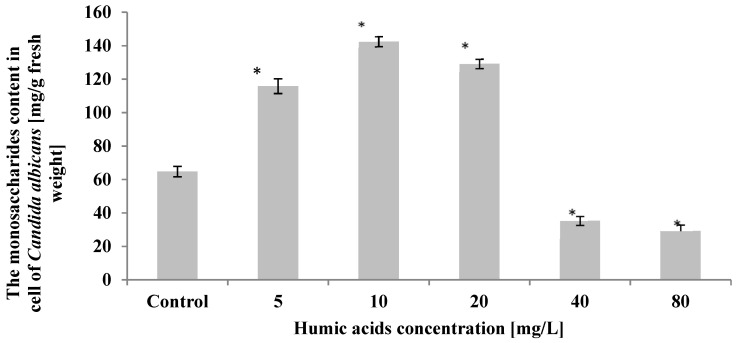
The effect of humic acids on the monosaccharaides content in cell of *Candida albicans* in the 5th day of culture (*n* = 8, average values ± SD, *p* ≤ 0.01), (*) shows statistically significantly different values in comparison with control sample.

*C. albicans* cells treated with humic acid at 5 mg/L, 10 mg/L, and 20 mg/L showed a statistically significant (*p* ≤ 0.01) increase in simple sugars compared to control. The highest 2-fold increase in these metabolites compared to control was shown in yeast cells treated with humic acid at 10 mg/L. In contrast, a decrease in monosaccharide content in yeast cells relative to the control was observed in cultures where the initial acid concentration was 40 mg/L and 80 mg/L. The monosaccharide content in *C. albicans cells* then decreased by 46–55% compared to control (Figure 4).

**Figure 5:**
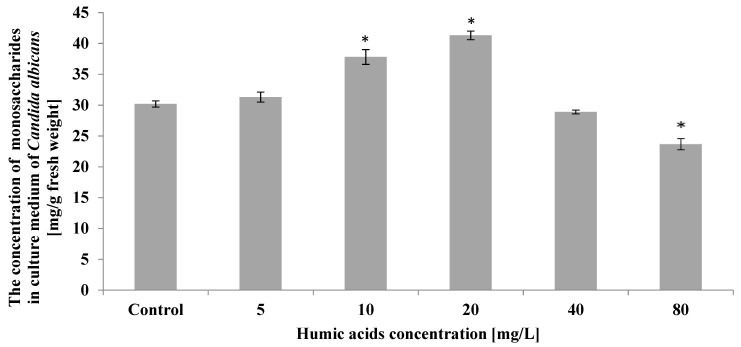
The effect of humic acids on monosaccharaide concentration in culture medium of *Candida albicans* on the 5th day of culture (*n* = 8, average values ± SD, *p* ≤ 0.01), (*) shows statistically significantly different values in comparison with control sample.

The experiment showed that monosaccharides secreted by *Candida albicans* cells into the medium increased in the presence of 10 mg/L and 20 mg/L humic acid. However, at 80 mg/L of humic acid, the simple sugars secreted by *C. albicans* decreased by 22% compared to control (Figure 5).

**Figure 6:**
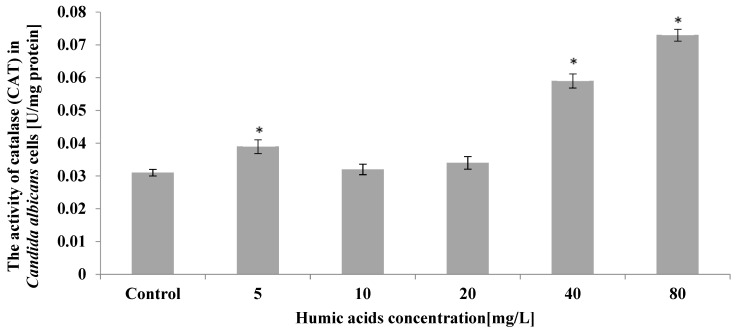
The effect of humic acids on activity of catalase (CAT) in *Candida albicans* cells on the 5th day of culture (*n* = 8, average values ± SD, *p* ≤ 0.01), (*) shows statistically significantly different values in comparison with control sample.

Humic acid at a concentration of 40 mg/L caused an increase in catalase activity in *C. albicans* cells by 90% compared to the control culture. On the other hand, the largest, more than 2-fold compared to the control, increase in catalase activity was recorded in the culture, where the initial concentration of humic acid was 80 mg/L. A slight increase in catalase activity was also noted in *C. albicans* exposed to humic acid at 5 mg/mL (Figure 6).

**Figure 7:**
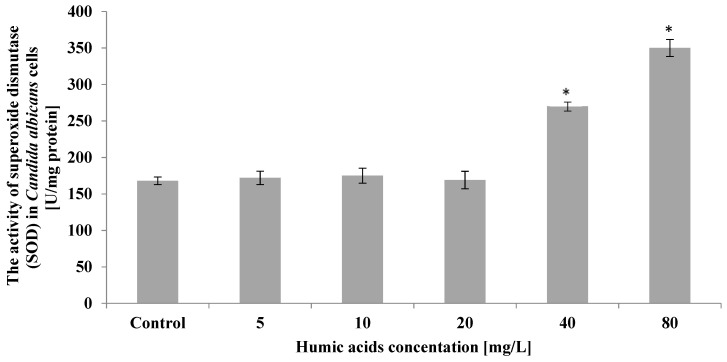
The effect of humic acids on activity of superoxide dismutase (SOD) in *Candida albicans* cells on the 5th day of culture (*n* = 8, average values ± SD, *p* ≤ 0.01), (*) shows statistically significantly different values in comparison with control sample.

Analyses showed insignificant differences, compared to the control, in superoxide dismutase activity in *C. albicans* cells treated with humic acid at 5–20 mg/L. In the culture in which the starting humic acid was 40 mg/L, a significant increase in superoxide dismutase activity was observed, by 61% relative to the control. The highest increase in the activity of this enzyme, more than 2-fold compared to the control, was observed in the culture treated with 80 mg/L humic acid (Figure 7).

## 4. Discussion

The conducted study showed a significant increase in the biomass of *Candida albicans* yeast exposed to exogenous humic acid at 5–20 mg/L compared to control. This indicates that this species is able to grow in waters rich in organic matter. The presence of *Candida albicans* is mainly observed in freshwater ecosystems, especially those with high levels of organic matter [22,30,31]. The observed increase in microfungal biomass exposed to humic acid may be associated with the specific structure of these compounds, abundant in aromatic compounds including phenols [32]. Aquatic fungi constitute a numerous group of destructive fungi that, thanks to hydrolytic enzymes, are able to decompose organic matter that other microorganisms cannot [33,34,35]. One such extracellularly secreted enzyme is manganese peroxidase, responsible for catalyzing reactions involving the oxidation of manganese from the +II oxidation state to the +III oxidation state. This change in manganese reactivity enables the efficient degradation of aromatic compounds [32]. 

In this study, high concentrations of humic acid in the range of 40–80 mg/L caused a significant reduction in *C. albicans* biomass. A similar effect has also been observed for prokaryotic organisms such as cyanobacteria [4,36]. It seems that this can be attributed to the high reactivity of humic substances associated with the presence of numerous functional groups such as carboxyl, carbonyl, amine, imidazole, phenolic, and alcohol. In the case of cyanobacteria, this is associated with blocking of the photosystem II and blocking the flow of electrons, thereby leading to a blockage of photosynthesis. It is very likely that an identical mechanism takes place in mitochondria, where electron transport processes also take place. This blockage results in reduced ATP production, leading to the generation of intracellular oxidative stress and thus to apoptosis of the fungal cell. At high concentrations of humic substances in water, *C. albicans* activates cellular defense mechanisms, as evidenced by the increase in antioxidant enzymes observed in this study. Moreover, the biomass of *C. albicans* increases at lower humic acid concentrations in water. Considering this, it seems that this yeast may be a good microbiological indicator of surface water pollution. 

The ability of *C. albicans* to thrive in waters rich in organic matter is likely the result of the fact that humic acid stimulates an increase in protein content in yeast cells, as well as simple sugars. The availability of organic matter induces cell division in microorganisms, which is accompanied by intensive protein biosynthesis in *C. albicans* cells. Gim and Kim [37] and Kulikova et al. [38] found that humic acid is able to stimulate aquatic organisms, increasing both biomass and metabolic rate. Another study on aquatic organisms in dystrophic lakes found that hydrobionts living in such waters may also have an increased carbohydrate metabolism [39]. Moreover, humic acid is one of the factors involved in causing gene expression [40]. High concentrations of humic acid are able to reduce the content of protein and simple sugars in yeast cells, which is consistent with literature data that high concentrations of humic substances have negative effects on aquatic organisms, creating stress conditions that can lead to lipid peroxidation and can initiate carcinogenesis [3,4]. 

The increase in monosaccharide secretion in our study may be a result of *Candida albicans* using sugars for adhesion. The sugar–protein complexes extracellularly form a covalent bond to the chitinous cell wall of the yeast to form a β-1.3-glucan-chitin bond, which facilitates yeast cell adhesion to various surfaces and biofilm formation [41]. Humic acid may have also acted as a stress factor on the fungal cells resulting in the destruction of the cell wall, which is approximately 40% composed of carbohydrates, leading to the release of sugars into the culture medium. In addition, one of the defense mechanisms of fungi in response to stress is the synthesis of trehalose, a sugar that is thought to protect the cell from adverse environmental conditions. The hydroxyl groups of trehalose bind to membrane proteins via hydrogen bonds causing stabilization of the cell. This carbohydrate is produced by, among other sources, the alcohol-fermenting yeast Saccharomyces cerevisiae, to defend itself against the negative effects of the high alcohol concentrations [42]. Saccharomyces cerevisiae exposed to a stress factor is able to synthesize heat shock proteins Hsp that protect the structural proteins of the cell from denaturation [43]. This may explain the high sustained protein concentration in the extracellular secretion of C. albicans exposed to humic acid.

C. albicans cells exposed to exogenous humic acid at 40–80 mg/L showed a more than twofold increase in catalase and superoxide dismutase activity compared to control. This increase may be due to the stress induced by humic acid, associated with the induction of oxygen radical synthesis and leading to lipid peroxidation, thus resulting in the production of anion radical which damages cell structures [4]. The cells produce superoxide dismutase, which breaks down the anion radical to hydrogen peroxide and oxygen in a two-step reaction. Hydrogen peroxide is also naturally produced as a byproduct of cell metabolism, but is toxic due to its high reactivity, and is easily diffused through cell membranes, causing damage to proteins, sugars, lipids and nucleic acids. Glutathione peroxidase is responsible for the neutralization of low concentrations of hydrogen peroxide, whereas when its concentration increases due to the action of superoxide dismutase, catalase decomposes peroxide by breaking it down to water and oxygen. The activity of both enzymes is highly correlated with each other because the enzymatic activity of superoxide dismutase is inhibited by hydrogen peroxide, while superoxide anion radical causes inhibition of catalase activity [44]. In addition, the humic acid-induced increase in the activity of antioxidant enzymes may be related to the increase in the sugars from fungal cells, as sugars play a defensive role against stress factors [45].

At a low concentration of humic acid (5 mg/mL), a slight but statistically significant increase in catalase activity was observed, while the SOD activity remained at the control level. This indicates that naturally occurring metabolic processes generate the appearance of hydrogen peroxide in fungal cells and that catalase is the main antioxidant enzyme of *Candida albicans* [46]. The highest concentrations of humic acid (5 and 10 mg/mL) were those concentrations that stimulated yeast growth and metabolism (especially protein and monosaccharide synthesis). The activity of neither enzyme increased significantly in comparison to the control culture, because anabolic over catabolic processes probably begin to prevail in the cells of fungi.

## 5. Conclusions

The results of the conducted research indicate that humic acid significantly influences the growth and metabolism of *Candida albicans*. At low concentrations (10–20 mg/L), humic acid caused an increase in the biomass and compactness of proteins and monosaccharides, in fungal cells, and in the extracellular secretion of the yeast. This may indicate that *Candida albicans* plays an important role in the microbial loop. Moreover, the increase in protein content in C. albicans cells combined with an increase in the activity of antioxidant enzymes may indicate that this yeast excels in conditions of high water pollution with organic matter of low bioavailability. This seems to be another important reason to include *C. albicans* as an indicator in the assessment of the ecological, toxicological, and sanitary status of surface waters, especially since many species of aquatic fungi are potential pathogens. Moreover, the high content of organic matter in the waters increases the mobility of other pollutants in the groundwater, including heavy metals.
